# Assessing the reliability of the FricTest^®^ 4.0 for diagnosing symptomatic dermographism

**DOI:** 10.1002/clt2.70005

**Published:** 2024-11-13

**Authors:** Annika Gutsche, Martin Metz, Melba Munoz, Kit Wong, Ted Omachi, Rui Zhao, Marcus Maurer, Vasiliki Zampeli, Markus Magerl

**Affiliations:** ^1^ Institute of Allergology Charité – Universitätsmedizin Berlin Corporate Member of Freie Universität Berlin and Humboldt‐Universität zu Berlin Berlin Germany; ^2^ Fraunhofer Institute for Translational Medicine and Pharmacology ITMP, Immunology and Allergology Berlin Germany; ^3^ Genentech Inc., 1 DNA Way South San Francisco California USA

To the Editor,

Symptomatic dermographism (SD), a common subtype of chronic inducible urticaria (CIndU), involves transient, strip‐shaped wheals that itch and burn when the skin is stroked or scratched.^1^ SD affects ≥0.5% of the population,^2^ yet despite its high frequency and marked impact on quality of life, diagnostic tools and treatment options are limited.^1^, ^3^

Diagnosis is based on the patient's medical history and provocation testing.^2^ Historically, provocation testing used a smooth, blunt object to stroke the skin, but variations in individuals' disease presentation highlighted the need for validated, reproducible tools.^3^ The FricTest®4.0^4^ is a hand‐held, flat plastic comb‐like tool with four smooth pins (3.0–4.5 mm long) firmly stroked along the skin. The resulting wheals determine the critical friction threshold (CFT), the shortest pin length/minimum pressure that elicits a positive wheal response.^5^

This study aimed to assess the reliability (reproducibility and repeatability) of FricTest inter‐rater agreement (results from two different raters on the same patient at the same visit) and intra‐rater agreement (results from the same rater on the same patient 7–14 days apart). Reliable results are important for monitoring treatment effects, helping patients understand triggers, and improving management. We assume that each patient's left and right forearms are the same, that visits are the same, and that previous provocation did not affect the reaction of subsequent tests.

This single‐center study was conducted at the Urticaria Center of Reference and Excellence^6^ at the Charité Hospital, Berlin, Germany. Adults had SD for >6 weeks, had active SD at enrollment, and gave written informed consent. The study followed the Declaration of Helsinki principles, and the Berlin Charité Ethics Committee approved the protocol.

The primary endpoint, inter‐rater agreement, was the intraclass correlation coefficient (ICC) between the CFT assessments of two raters within the same patient. The CFT scale is 0–4 (0 = no response, 4 = maximum response). ICCs plus upper and lower 95% confidence intervals (CI) were calculated using a mixed‐effects linear model methodology.^7^ Rater A and B agreement was quantified using weighted kappa across four categories: left forearm, right forearm, Visit 1, and Visit 2. An ICC<0.4 indicated poor reliability, and 0.6–0.8 indicated substantial reliability.

Two raters randomized to the order of assessments (Forearm 1 [right], Forearm 2 [left]) administered and recorded all FricTest results. At Visit 1, Rater A applied the FricTest to Forearm 1, covered the arm, and left the room. Rater B then applied the FricTest to Forearm 2, covered the arm, and left the room. Ten minutes after application one, Rater A returned, uncovered Forearm 1 and documented the reaction before re‐covering the arm. Rater B repeated this process. Both raters repeated these procedures at Visit 2 after 7–14 days, using the opposite arm to Visit 1.

Among 16 participants (62.5% female), we observed substantial inter‐rater agreement when Rater A and B assessed the same patient during the same visit, with a weighted kappa of 0.86 for the left and 0.77 for the right forearm (Table 1 **,** Figure 1). When the two raters assessed the same patient at different visits, the agreement was moderate at Visit 1 (weighted kappa of 0.56) but improved to substantial at Visit 2 (0.79; Table 1).

**TABLE 1:** Inter‐rater and intra‐rater agreement.

	Rater/Arm/Visit	Weighted kappa
Inter‐rater agreement: Rater A versus B		
Left arm		0.86
Right arm		0.77
Visit 1		0.56
Visit 2		0.79
Intra‐rater agreement: A versus A, B versus B		
Left arm versus right arm	A	0.72
	B	0.73
Visit 1 versus visit 2	A	0.72
	B	0.73

**FIGURE 1:**
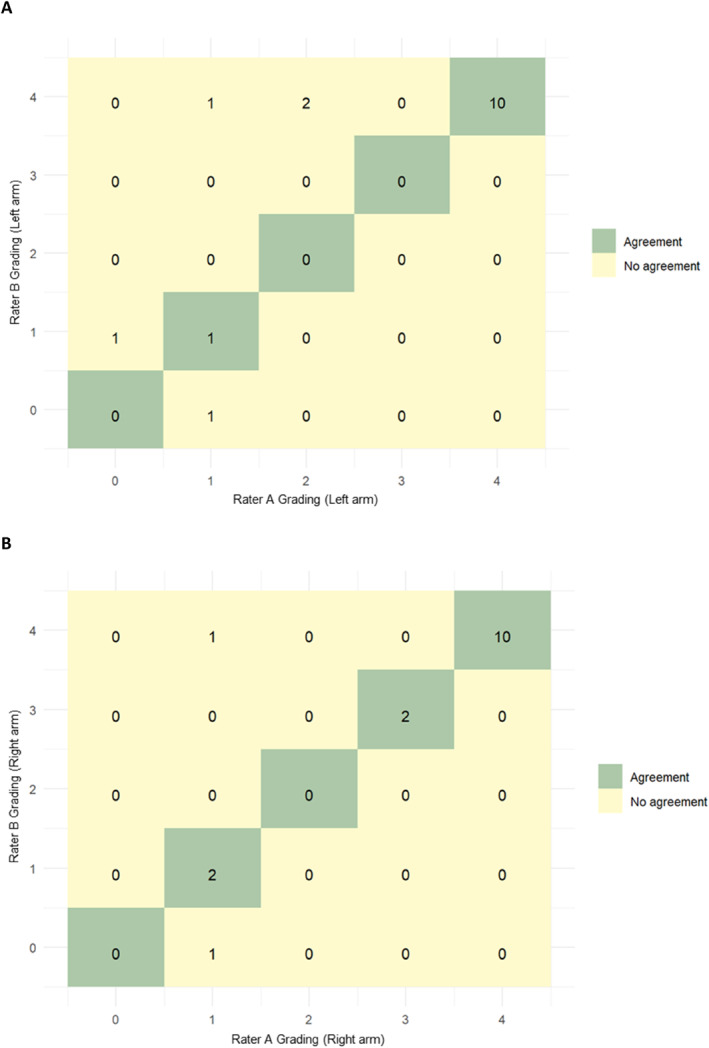
In each plot (A and B), the *x*‐axis is represented by the grading from Rater A, and the *y*‐axis is the grading from Rater B for the left (A) and the right forearm (B). Both axes range from 0 to 4. The matrix numbers indicate where Rater A and B provided identical gradings (agreement is marked in green) or different gradings (no agreement is marked in yellow) for the scores. For example, as indicated in (A), Rater A and B assessed the left arm as agreeing with FricTest scores in 11 of 16 patients.

When the same rater assessed one patient at different visits, we found substantial intra‐rater agreement. Raters A and B reported weighted kappas of 0.72 and 0.73, indicating consistent and substantial agreement between Visits 1 and 2.

Overall, there was high consistency in ICC measurements, with 0.89 (95% CI 0.71, 0.94) for inter‐rater reliability and 0.81 (95% CI 0.60, 0.92) for intra‐rater reliability. At Visits 1 and 2, the average mean differences between Rater A and B were 0.06 and 0.13, respectively. Similarly, the average mean differences for the left and right forearms were 0.06 and 0.25, respectively, indicating high agreement essential for reliable measurements.

Our study validates the FricTest, demonstrating its reliability as a diagnostic and monitoring tool in SD. Measurement noise from repeated tests by the same or different raters is a well‐recognized source of error in medical assessments.^8^ Our results showed almost perfect inter‐rater and substantial intra‐rater agreement when inducing wheals in SD patients. Accurately determining a patient's CFT could enhance SD diagnosis and ultimately lead to better management of the patient's condition.

We observed changes in one patient that primarily drove variability between visits. Despite these fluctuations, the consistency with which each rater detected changes in disease activity confirms the robustness of our assessment tool in identifying improvements and deteriorations in SD.

Our study has limitations, mostly relating to its small size, duration of follow‐up, and applicability to real‐world conditions. Itch was documented at baseline to ensure the correct diagnosis; it could not be measured as a result because the intensity of itch cannot be differentiated in such tightly condensed strips. Further real‐world evidence needs to confirm the FricTest's reproducibility over longer periods and clinical validity in busy settings, which differ from the controlled conditions within this study. While we strove to ensure comparable physical environments at both visits, we cannot rule out minor differences in the experimental environment nor exclude disease‐activity modifying factors between the two visits, which may have caused response fluctuations.

The simple, compact and affordable FricTest should be utilized in clinical studies, especially those investigating potential CIndU treatments, and in primary care settings, where continuity of care can be challenging.

## AUTHOR CONTRIBUTIONS

**Annika Gutsche**: Methodology; writing—review & editing; writing—original draft; data curation. **Martin Metz**: writing—original draft; methodology; writing—review & editing; data curation. **Melba Munoz**: Writing—original draft; methodology; writing—review & editing; data curation. **Kit Wong**: Methodology; writing—review & editing; data curation. **Ted Omachi**: Data curation; methodology; writing—review & editing. **Rui Zhao**: Methodology; data curation; writing—review & editing. **Marcus Maurer**: Methodology; writing—original draft; writing—review & editing; data curation. **Vasiliki Zampeli**: Study protocol; study investigator; methodology; writing—review & editing; writing—original draft; data curation. **Markus Magerl**: Study protocol; study investigator; writing—original draft; methodology; writing—review & editing; data curation.

## CONFLICT OF INTEREST STATEMENT

**A. Gutsche** declares no conflict of interest**. M. Metz** is or recently was a speaker and/or consultant for Amgen, AstraZeneca, Argenx, Celldex, Celltrion, Escient, Jasper Therapeutics, Novartis, Pharvaris, Regeneron, Sanofi, and Third Harmonic Bio. **M. Munoz** is or recently was a speaker, advisor and/or received research funding from Jasper Therapeutics, Celldex Therapeutics, Takeda, GA^2^LEN, UNEV, Astra Zeneca and Roche outside the submitted work. **K. Wong** is a former employee of Genentech Inc. **T. Omachi** is an employee of Genentech Inc. **R. Zhao** is a former employee of Genentech/Roche. **M. Maurer** was a speaker and/or advisor for and/or has received research funds from Allakos, Amgen, Aralez, ArgenX, AstraZeneca, Celldex, Centogene, CSL, Behring, FAES, Genentech, GI Innovation, Innate Pharma, Kyowa Kirin, Leo Pharma Lilly, Menarini, Moxie, Novartis, Roche, Sanofi/Regeneron, Third Harmonic Bio, UCB, and Urich. **V. Zampeli** was a speaker and/or advisor for and/or has received research funding from Pharming, Takeda, and CSL Behring. **M. Magerl** is or recently was a speaker and/or advisor for and/or has received research funding from Biocryst, Pharming, Takeda, CSL Behring, Ionis Pharmaceuticals, KalVista, and Pharvaris.
